# The Surgical Complications and Sequels of Typhoid Fever

**Published:** 1899-12

**Authors:** 


**The Surgical Complications and Sequels of Typhoid Fever.** By WILLIAM W. KEEN, M.D. Pp. 386. London: The Rebman Publishing Co., Ltd. 1898.

This book is a monument of patient and laborious research. It contains in the form of an appendix a reprint of the author's "Toner Lecture" delivered in 1876, "On the Surgical Complications and Sequels of the Continued Fevers," and in addition it contains the substance of a lecture delivered by the author in 1896, or just twenty years after the former lecture, embracing the literature on the subject which had accumulated during the interval—an important interval for such a topic, inasmuch as it introduced the discovery of the typhoid bacillus of Eberth, and the development of modern surgery in respect to opening the abdomen for the relief of perforations and other lesions arising in the course of typhoid fever. The author, in conjunction with Dr. Thompson S. Westcott, has collected no less than 1700 cases, which practically include nearly all the cases recorded in the last fifty years, and he expresses his obligation to the gentlemen in charge of the library of the Surgeon General's Office, U.S.A., for placing at his disposal "the treasures of that unrivalled collection. . . . The enlightened and liberal management of that library has made the whole world debtors to America," and such books as the one before us would be impossible without such an institution.

Dr. Keen commences with a good account of the pathology of typhoid fever, sufficiently full to show its bearing from the surgical side; thus such subjects as the viability of the typhoid bacilli both outside and inside the human body, and their wide diffusion in various organs and tissues of the body, are fully considered. He next describes in full detail the various surgical complications, such as affections of the bones, joints, blood-vessels and other organs, gangrene, abscesses, and, chief of all, perforations of the intestine and gall-bladder. In the case of perforations of the intestine, he gives a table of 83 operations, collected by Dr. Westcott for this work. Of these 83 cases, 16 recovered, or 19.36 per cent. of cures and 80.64 per cent. of deaths. When this is contrasted with Murchison's figures of 90 to 95 per cent. of deaths after perforation without operation, we may (as Dr. Keen remarks) well take courage for the future.

This book will certainly be the standard work on the subject for some years to come. It has evidently been prepared with great care and attention to detail, and should be studied by all surgeons who are liable to be called upon to treat complications occurring during or after typhoid fever.

